# Replication and Refinement of an Algorithm for Automated Drusen Segmentation on Optical Coherence Tomography

**DOI:** 10.1038/s41598-020-63924-6

**Published:** 2020-04-30

**Authors:** Maximilian W. M. Wintergerst, Shekoufeh Gorgi Zadeh, Vitalis Wiens, Sarah Thiele, Steffen Schmitz-Valckenberg, Frank G. Holz, Robert P. Finger, Thomas Schultz

**Affiliations:** 1 Department of Ophthalmology, University Hospital Bonn, Ernst-Abbe-Str. 2, 53127 Bonn, Germany; 2 Department of Computer Science, University of Bonn, Endenicher Allee 19a, 53115 Bonn, Germany; 3 Department of Medical Biometry, Informatics and Epidemiology, University of Bonn, Bonn, Germany; 4 TIB Leibniz Information Centre for Science and Technology, Hannover and Fraunhofer IAIS, St. Augustin, Germany; 5 Bonn-Aachen International Center for Information Technology, University of Bonn, Endenicher Allee 19a, 53115 Bonn, Germany

**Keywords:** Biomarkers, Translational research, Macular degeneration, Retinal diseases

## Abstract

Here, we investigate the extent to which re-implementing a previously published algorithm for OCT-based drusen quantification permits replicating the reported accuracy on an independent dataset. We refined that algorithm so that its accuracy is increased. Following a systematic literature search, an algorithm was selected based on its reported excellent results. Several steps were added to improve its accuracy. The replicated and refined algorithms were evaluated on an independent dataset with the same metrics as in the original publication. Accuracy of the refined algorithm (overlap ratio 36–52%) was significantly greater than the replicated one (overlap ratio 25–39%). In particular, separation of the retinal pigment epithelium and the ellipsoid zone could be improved by the refinement. However, accuracy was still lower than reported previously on different data (overlap ratio 67–76%). This is the first replication study of an algorithm for OCT image analysis. Its results indicate that current standards for algorithm validation do not provide a reliable estimate of algorithm performance on images that differ with respect to patient selection and image quality. In order to contribute to an improved reproducibility in this field, we publish both our replication and the refinement, as well as an exemplary dataset.

## Introduction

To increase our understanding of risk factors for age-related macular degeneration (AMD), the leading cause of irreversible blindness in the developed world, large, prospective epidemiological studies on AMD retinal biomarkers are warranted^1–4^. Increasing amounts of optical coherence tomography (OCT) data are being generated and need to be assessed. However, as the manual grading of enormous data volumes is unfeasible automated algorithms for OCT image analysis are needed^5,6^. To date a variety of different algorithms on quantitative OCT image analysis for AMD biomarkers including drusen, geographic atrophy, pigment epithelial detachment and intra- and subretinal fluid have been published^7,8^. However, algorithm quality and performance differ substantially making comparison between algorithms challenging^8,9^.

When selecting an algorithm from the literature, one for which high accuracy has been reported would be preferable. However, this does not guarantee comparable performance on one’s own data. In particular, accuracy depends not only on the algorithm itself, but also on the characteristics of the dataset. Frequently, privacy and legal reasons make it impossible to openly share the data on which algorithms have been evaluated. At the same time, only very few authors make their software implementations available. Even though proper validation of an algorithm should involve testing it on data that was not available during its development, and characterizing conditions under which it can be expected to work well, the reasons above make this difficult within the field of medical image analysis.

The current best practice for validating medical image analysis algorithms is to organize so-called “challenges”, in which different teams apply their algorithms to a common reference image data set. In regards to OCT image analysis both the “Retinal OCT Classification Challenge (ROCC)”^10^ and the MICCAI “Retinal OCT Fluid Challenge (RETOUCH)”^11^ are available and have been used for some of the respective algorithms published. Challenges are an important tool for algorithm validation, but their open nature implies incomplete coverage, as promising approaches might be missing because their authors chose not to participate. Moreover, it has been found that reproducibility and interpretation of their results is limited by the fact that participants are given the opportunity to adapt their methods to the data at hand, but often have to do so based on insufficient information. Challenges have also been shown to rank algorithms in a way that depends not only on their quality, but also substantially on contestable choices in the ranking scheme^12^. Therefore, we believe that replication studies, which received almost no attention in the literature so far, should complement challenges as another tool for algorithm validation.

We conducted such a replication study. Specifically, we re-implemented an algorithm for drusen quantification on OCT proposed by Chen *et al*.^13^, which we identified as an established algorithm that was reported to achieve strong results in a previous systematic literature review^8^. Following assessment of its performance, we refined this algorithm to better meet the characteristics of our data. Our results highlight the severe limitations that incomplete algorithm validation poses for practical use.

## Methods

### Image data acquisition

The patient sample is a random subsample from the “Molecular Diagnostics of Age-related Macular Degeneration” (MODIAMD) study from the University of Bonn, Germany (Federal Ministry of Education and Research funding number 13N10349)^14^. Briefly, inclusion criteria were age >50 years and retinal alterations classified as Age-Related Eye Disease Study (AREDS) category 3 or 4. Exclusion criteria for the MODIAMD study were any other ophthalmic disease potentially comprising the assessment of the retina as was concomitant injection-therapy for AMD. All study subjects consented to participate in the study. The tenets of the Declaration of Helsinki were followed and this study was approved by the ethics committee of the University of Bonn, Germany (ethics committee number: 175/10 and 408/15). SD-OCT raster scans were acquired using the Spectralis HRA + OCT (Heidelberg Engineering, Heidelberg, Germany) with a field size of 20° × 15° centered on the fovea and an OCT image resolution of 512 × 496. A representative subset of 81 volume scans each with 145 B-scans consisting of at least 15 averaged frames and with an approximate inter-B-scan distance of 30 μm was used for this study. These scans were selected from a larger set of 682 OCT volume scans from 98 patients, keeping about one scan per patient in order to cover a wide range of image quality and drusen phenotypes. Those volumes that had very few B-scans or insignificant drusen load were discarded from the final subset. Geographic atrophy was defined according the cRORA criteria^15^.

### Replication of Chen *et al*. algorithm

As described in the work of Chen *et al*.^13^ several techniques are used to denoise the image, remove the retinal nerve fiber layer (RNFL) and compute the centerline of the retinal pigment epithelium (RPE) (Supplemental Figs. 1 and 2). In their algorithm, Chen *et al*. first denoise the input, using a bilateral filter with an anisotropic window to account for the stretch of B-scans in the horizontal direction. Then B-scans are binarized using a threshold 0.3*t to detect and remove the RNFL layer, and with threshold t in order to detect ellipsoid zone and the RPE. The center-line of the estimated layer is considered as the final estimation of the RPE. In order to detect drusen, a 3^rd^ degree polynomial is fit on the RPE layer, estimating a drusen-free RPE. The area between the drusen-free RPE and RPE layer are considered as drusen. After detecting drusen per B-scan, the en face OCT image was used for a false-positive elimination-step as proposed by Chen *et al*.^13^ (Supplemental Fig. 3). The details of this algorithm, as well as the data-specific tuning needed to adjust the algorithm for the MODIAMD data-set, is provided in the supplementary materials.

We observed that, in Chen’s approach, inclusion of parts of the ellipsoid zone along the RPE can lead to jumps in the estimated RPE layer, as shown in Supplemental Fig. 4 (false positives). Therefore, our work suggests further refinements for a more robust determination of the RPE layer.

### Refinement of the algorithm

Our refined algorithm reduces the above described false positives by better separating ellipsoid zone and RPE. In particular we found that shadows can cause the brightness of the RPE to vary substantially within B-scans, as it can be seen in Supplemental Fig. 4. In these cases, it is less suitable to use a global threshold for RPE segmentation. Therefore, we propose three refinement steps for thresholding, where the segmentation mask of each step is used as a guide to remove irrelevant components of the mask that is computed at the next step. We also replace the bilateral filter, which is used for denoising by Chen *et al*., with the multi-scale anisotropic fourth-order diffusion (MAFOD) filter^16^. This filter was developed specifically to enhance ridges, which helps better localizing the center-line of the estimated RPE layer (Supplemental Fig. 5).

**Figure 1:**
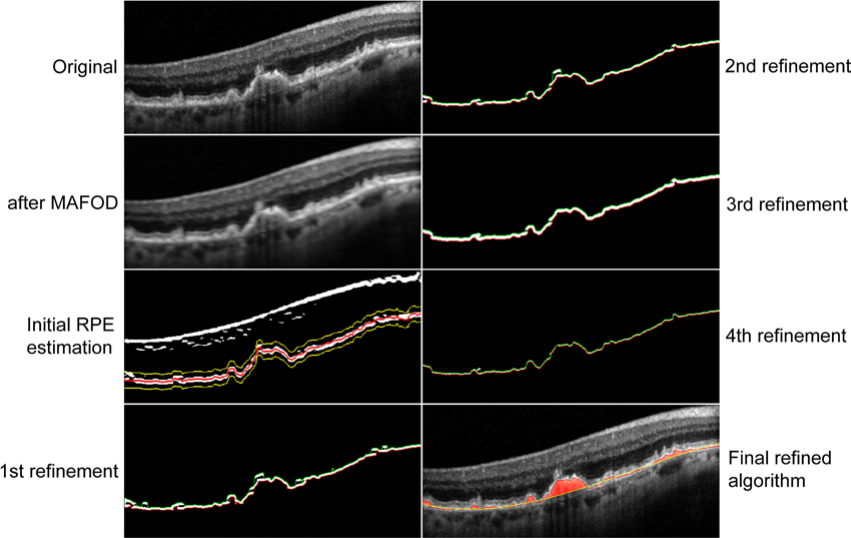
Step-by-step illustration of the refinements.

In the first refinement step (Supplemental Fig. 6), we perform the thresholding with respect to both denoised input B-scan and a local histogram equalization of the input B-scan. In the second refinement step (Supplemental Fig. 7), a threshold with respect to the local histogram equalization of the denoised image, and the denoised image itself is selected to binarize the B-scan. In the third refinement step (Supplemental Fig. 8), a Gaussian blur is applied on the denoised image. Then the same steps as in the second refinement step are applied on the smoother version of the denoised B-scan. Finally, in the fourth refinement step, we implemented an improved method for estimating the lower and upper boundaries of the RPE from the segmentation mask by boundary tracing and polynomial fitting (Supplemental Fig. 9). We take the center line between the upper and lower boundary as the final refined estimation of the RPE layer. Figure 1 illustrates the different refinements step-by-step on one B-scan from the original image till the final refined algorithm. More details on the refined algorithm can be found in the supplementary materials.

The input B-scan (left, first row) is filtered with MAFOD filter (left, second row). The retinal pigment epithelium (RPE) is estimated, twofold enlarged and threshold-positive pixels outside a 20-pixel band around the RPE centerline (left, third row; red and yellow indicate the centerline and the 20-pixel band, respectively) are removed (left, last row; green and red indicate the upper and lower boundary of the RPE, respectively). The second, third and fourth refinement steps are applied (right, first, second and third row; green and red indicate the upper and lower boundary of the RPE, respectively) to achieve the final refined algorithm (right, last row). See supplement for details.

## Results

In exact analogy to Chen *et al*.^13^, algorithm performance was quantitatively evaluated by the absolute drusen area difference (ADAD) and overlap-ratio in relation to the ground truth. Similar to Chen *et al*. we evaluated the algorithm’s performance for a dataset where only B-scans with drusen were included and for a subset where only the B-scan with most drusen load in each volume scan was included^13^. Original results from Chen *et al*. are displayed in Table 1 and our results of the replicated algorithm are reported in Table 2. Performance of the replicated algorithm was inferior to the reported original algorithm and performance of the refined algorithm was superior to the replicated one.

**Table 1:** Original results from Chen *et al*.^13^.

	ADAD [μm]	ADAD [%]	OR ± SD
B-scans with drusen (‘4/340 dataset’)	10.29 ± 8.9	15.70 ± 15.50	76.33 ± 11.29
B-scans with largest drusen load per volume (‘143/143 dataset’)	19.97 ± 14.68	23.77 ± 13.8	67.18 ± 9.14

Image resolution of the used dataset: 512 × 1024 and 128 B-Scans per volume scan; OR = overlap ratio; SD = standard deviation.

**Table 2:** Comparison of the algorithms to the ground truth.

	Replicated Chen *et al*.^13^			Refined algorithm		
	ADAD ± SD (μm)	ADAD ± SD (%)	OR ± SD (%)	ADAD ± SD (μm)	ADAD ± SD (%)	OR ± SD (%)
B-scans with drusen	17.60 ± 36.70	100.59 ± 304.80	24.52 ± 20.56	13.28 ± 29.40	73.54 ± 217.80	35.88 ± 25.25
B-scans with largest drusen load per volume	19.94 ± 13.54	42.70 ± 22.70	39.24 ± 22.06	15.64 ± 11.05	36.86 ± 24.34	51.90 ± 23.70
Volumetric Computation	11.96 ± 12.11	46.37 ± 75.36	29.35 ± 17.32	8.31 ± 6.87	30.05 ± 29.76	42.20 ± 20.47

Image resolution of the used dataset: 512 × 496 and 145 B-Scans per volume scan. OR = overlap ratio; SD = standard deviation.

Even though we present the same error metrics as used by Chen *et al*. in order to facilitate a direct comparison, we noticed two limitations in the way they are defined. First, restricting the evaluation to B-scans in which drusen are present might conceal some of the false positives that occur in drusen-free B-scans. However, these are practically relevant, since we want to employ the algorithm fully automatically, without having to flag drusen present B-scans manually. Second, overlap ratio is a relative error metric, as is ADAD when expressed in percent. Therefore, computing based on B-scans can result in inflated estimates in the presence of B-scans with low drusen load, since even small absolute segmentation errors will correspond to a large relative error. We also note that based on the information given in the publication by Chen *et al*.^13^ it is not completely clear how overlap ratio was aggregated, it is merely stated to be “similar as for the ADAD metrics”.

For these reasons, Table 2 presents an additional evaluation, referred to as “volumetric computation”, which is based on the full OCT volumes. Given a three-dimensional ground truth drusen mask *M*_*i*_ for the *i*th OCT volume, and a corresponding algorithmic estimate $\widehat{{M}_{i}}$, our volumetric computation error measures can be expressed as

$${\text{ADAD}}_{\text{i}}=|\text{Area}({M}_{i})-\text{Area}({\hat{M}}_{i})|$$

$$\text{Overlap}\,{\text{Ratio}}_{i}=\frac{\text{Area}({M}_{i}\cap {\hat{M}}_{i})}{\text{Area}({M}_{i}\cup {\hat{M}}_{i})}$$

where Area(*M*_*i*_) denotes the overall drusen area, summed over all B-scans of the *i*th volume. For error measure *E* ∈ {ADAD,OR}, mean and standard deviation are computed according to their established definitions:

$${\mu }_{E}=\frac{1}{N}\mathop{\sum }\limits_{i=1}^{N}{E}_{i},{\sigma }_{E}=\sqrt{\frac{1}{N}\mathop{\sum }\limits_{i=1}^{N}{({E}_{i}-{\mu }_{E})}^{2}}$$

These alternative error metrics differ from the ones used by Chen *et al*. in two main ways: First, they also account for false positives in drusen-free B-scans (there were 1,934 drusen-free B-scans in our dataset). Second, mean and standard deviation are taken over volumetric OCT scans rather than B-scans to avoid an inflated effect of B-scans with low drusen load on relative error measures. In non-volumetric measures as proposed in Chen *et al*., per B-scan ADAD is divided by the number of A-scans with drusen present in that B-scan. For a fair comparison between the volumetric ADAD measure to its non-volumetric alternatives, we divide ADAD by the sum of the A-scans with drusen for all B-scans in the volume.

**Figure 2:**
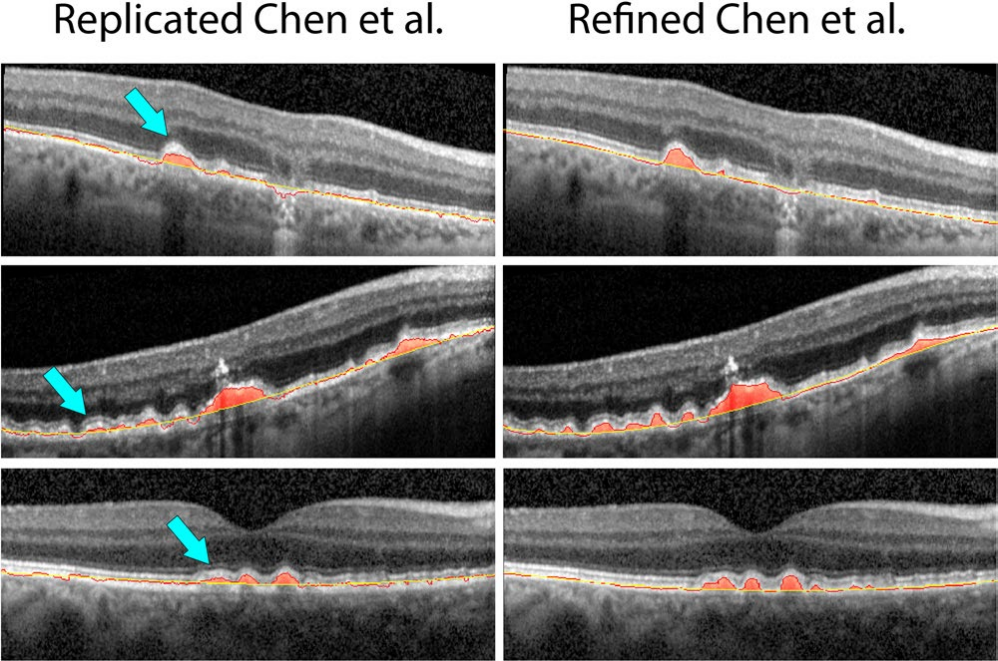
Comparison of replicated and refined drusen segmentation.

Separation of the RPE and the ellipsoid zone, which was a major source of segmentation errors in the replicated algorithm, could be improved by the refined algorithm. Our refinement of this algorithm was able to detect the course of the RPE-centerline more reliably (Fig. 2). This suggests that our refined algorithms allows for a more robust determination of the RPE layer than the replicated algorithm. When comparing the replicated and refined Chen *et al*. algorithm there were only two OCT volume scans where the replicated algorithm outperformed the refined one. It should be noted that algorithm performance was low for both algorithms in these two OCT volume scans (OR of 13% and 11% for the refined algorithm, and 14% and 15% for the replicated algorithm, respectively). Both of the OCT volume scans were from eyes with subretinal drusenoid deposits.

Results from the replicated Chen *et al*. method (left), and the refined method (right) marked over different exemplary input B-scans.

The mean drusen load of the complete dataset was 153,166,119 μm³ in the ground truth, 119,321,550 μm³ in the replicated algorithm, 125,837,159 μm³ in the refined algorithm. Hence, both algorithms underestimate drusen load, but our refined one less severely. To investigate the role of the false-positive elimination step for this underestimation, we compared total drusen calculation with and without false-positive elimination. We found that drusen load was underestimated even without false positive elimination (replicated algorithm: 132,385,230 μm³, refined algorithm: 132,555,984 μm³).

**Figure 3:**
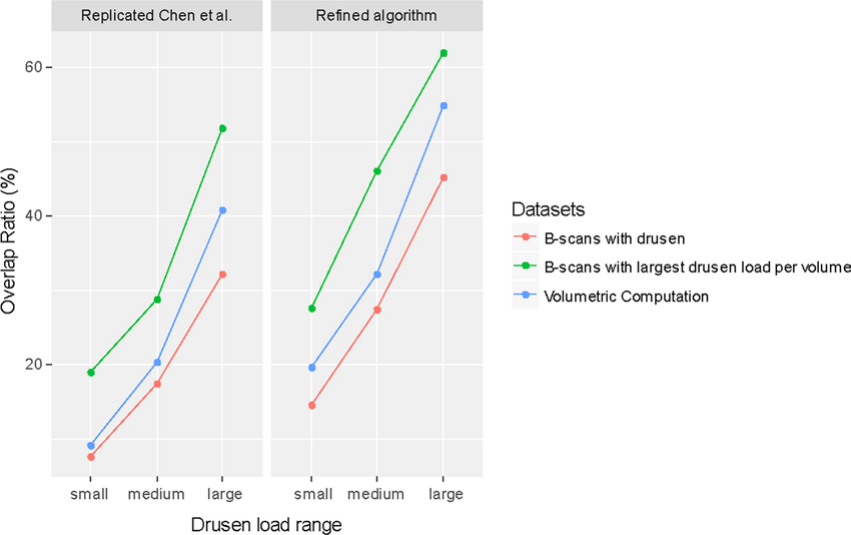
Algorithm performance stratified for drusen load.

In order to estimate the effect of the drusen load we stratified our results for small (0–26,465,894 μm³), medium (26,465,894–92,630,630 μm³) and large (>92,630,630 μm³) drusen load per volume scan (Fig. 3). Overlap ratio increased for all data subsets and algorithms with increasing drusen load.

To investigate the effect of geographic atrophy on algorithm performance, we compared results in B-scans with and without geographic atrophy, which was present in 17 of our OCT volume scans (21%). As presence of geographic atrophy is correlated with greater drusen load, and since we found algorithm accuracy depending on drusen load, we performed a multiple regression analysis for both algorithms with overlap ratio from our volumetric computation as the dependent variable and the binned drusen load categories “small”, “medium”, and “large” and presence of geographic atrophy as independent variables. The regression analysis showed a significant association with drusen load categories but not with geographic atrophy (see Supplemental Table).

### Statistical analysis

We used the Wilcoxon signed-rank test to evaluate the segmentation improvement using our proposed refined algorithm, with respect to ADAD measure in μm. The Shapiro-Wilk test on the ADAD values showed a non-normal distribution with the p-values of 6.9 × 10^−12^ for ADADs computed per OCT volume, p-value = 0 for B-scans with at least one druse, and p-value = 1.1 × 10^−8^ for B-scans with largest drusen load per volume. This motivated us to use a non-parametric version of the paired Student’s t-test, i.e., the Wilcoxon signed-rank test. With this test, the p-values for the paired samples using the replicated and refined Chen *et al*. algorithms are: 2.4 × 10^–6^ for ADADs computed over the complete OCT volumes, p-value = 0 for B-scans with at least one druse, and p-value = 1.2 × 10^−5^ for B-scans with largest drusen load per volume. These numbers show a significant segmentation improvement with respect to ADAD measure, when the refined Chen *et al*. algorithm is used.

### Risk of bias evaluation

Risk of bias for the new drusen segmentation algorithm was evaluated according to the standardized protocol as published previously^8^. The patient sample was recruited out of the MODIAMD data with inclusion criteria as stated above. The algorithm development and testing were performed in separated data subsets, the reference standard was a manually corrected segmentation of the RPE layer and the reference standard was objectively compared with the index test (via ADAD and overlap ratio). There were two ophthalmologists as readers for the annotations (ST and MW) and no repetitive measurements by these readers. All included patients received the same reference standard. Parameters increasing risk of bias are that subjects were not randomly recruited and there were no repetitive measurements done.

## Discussion

Re-building an algorithm based on the details provided in the publication we found its performance to be inferior to the reported results. This was likely due to a more heterogeneous dataset including a more real-life patient selection and a greater diversity in drusen load and differences in image quality. Following this, several refinements considerably improved the overall performance of the algorithm in our image dataset. These findings highlight that automated algorithms have to be used with caution, particularly when little or no independent evaluation and validation are available.

We identified multiple factors that might explain the discrepancy between our results and those reported by Chen *et al*., the most important being the patient cohort. Different from Chen *et al*., our study population included individuals with geographic atrophy, making it more heterogeneous and closer to a real-world situation. In addition, we conjectured that differences in drusen load might explain differences in performance. Against this background we performed a multiple regression analysis which showed no association of algorithm performance with presence of geographic atrophy, however a strong association with drusen load. Therefore, we hypothesize differences in drusen load might be an important factor for the observed difference in algorithm performance. However, this remains speculative as the absolute drusen load was not published by Chen *et al*. Another factor concerns image quality. The reduced axial resolution of 496 pixels compared to the input data of the Chen *et al*. algorithm which had an axial resolution of 1024 pixels is likely to reduce accuracy of the separation of RPE and ellipsoid zone and reduce performance somewhat. Although algorithm accuracy might further improve by exclusion of poor or reduced image quality, e.g. due to lens opacities, we did not apply any ancillary exclusion criteria additional to the MODIAMD exclusion criteria^14^, as our intention was to investigate algorithm performance in a dataset which is as close to ‘real-life’ conditions as possible.

These differences in patient and image characteristics are unavoidable, since the data used by Chen *et al*. is not available to others. Characteristics of OCT image data can differ greatly, e.g. due to differences between devices or in patient samples. Even when using the same device, differences in image acquisition are possible based on varying number of repetitive frames and consecutive image averaging, altered resolution or field-of-view settings, etc.. Against this background, it is even more important to provide highly specific details on both the algorithms as well as the image data used in their creation and evaluation. It also highlights the importance of testing algorithms on different datasets to achieve a full validation.

As an aside we proposed alternative or additional error metrics which might bear advantages over the so far used error metrics. However, none of our main results depend on the choice of error metric and in particular the improvement in algorithm performance following our refinements is independent of this.

As the refined Chen *et al*. algorithm still underestimated overall drusen load, one possible approach to reduce underestimation of drusen load might be an improvement of the polynomial fitting step. Both algorithms (as many others) use polynomial fitting for ideal RPE estimation. However in presence of large drusen or many small drusen, the estimated ideal RPE is ‘lifted up’, hence, leading to drusen underestimation.

There were only two OCT volume scans where the replicated algorithm outperformed the refined one. Overall performance was low for both OCT volume scans and both were from eyes with subretinal drusenoid deposits, rendering presence of subretinal drusenoid deposits a potential limitation of both the replicated and the refined algorithm. A possible next step would be to statistically compare algorithm performance in eyes with and without subretinal drusenoid deposits. In case this assumption is confirmed, further refinements might be introduced to specifically deal with subretinal drusenoid deposits.

Our results showed a lower ADAD in the volumetric computation metric compared to the subsets “B-scans with drusen” and “B-scans with largest drusen load per volume”. This observation can be explained by the fact that both algorithms underestimate overall drusen load. The volumetric computation metric adds the contribution of false positives in the drusen-free B-scans, which reduces this underestimation, and therefore leads to a smaller difference between estimated and actual drusen load. The fact that the volumetric computation metric increases overlap ratio compared to averaging over all B-scans with drusen, but not to B-scans with largest drusen load, can be explained by overlap ratio being a relative error measure. Therefore, its volumetric computation variant reduces the effect of B-scans with low drusen load, where even small absolute errors can cause a drastic reduction of overlap ratio.

Herein, we focussed on an established segmentation algorithm based on traditional image processing techniques such as filtering and thresholding. Recently, convolutional neural networks (CNNs) and deep learning have become more popular also for automated image analysis in ophthalmology^17,18^. We expect that issues of reproducibility and transferability will become even more relevant when using deep learning. Reproducing a learning based method involves not just re-implementation, but also re-training of the algorithm. Consequently, the used training datasets and their evaluation need to be highly transparent and reproducible. Moreover, CNNs’ results can be easily skewed by specific targeted manipulation of the input data, not recognizable by humans^19^. It is even possible to generate images completely unrecognizable to humans, which deep learning algorithms believe to be recognizable objects with >99% certainty^20^. This highlights some of the advantages of conventional, human-designed algorithms, where it is easier to rationalize the effects of factors such as reduced image resolution, and to ameliorate them with refinements such as those proposed in our current work.

To our knowledge, within the field of ophthalmic image analysis, we present the first study on re-implementing an algorithm based on the details provided in its publication, drawing attention to the important issues of algorithm reliability and replicability. Our results highlight that more details, both concerning the algorithm and the data that it is applied to, might be relevant for a proper replication than is typically given in a publication.

Further strengths of our study are the employment of a more real-life patient selection as dataset and a detailed step-to-step explanation of the algorithm’s refinement. Furthermore, we also made a comprehensive evaluation of algorithm performance, a direct comparison of the replicated and the refined algorithm in the same dataset and a subgroup analysis for drusen load. A limitation of our study is the reduced axial resolution as compared to the input data of the original algorithm. However, this reflects a more realistic real-life dataset.

In conclusion, we replicated a reportedly well-performing algorithm for OCT-based drusen quantification and found algorithm performance to be inferior to the reported results for various reasons. Several refinements considerably improved algorithm performance in our sample but still did not achieve published results. Replication of a published algorithm based on the details provided in a publication is challenging and better standards to ensure algorithm reproducibility, reliability and validity should be established as an increasingly large part of day to day clinical medicine is informed by automated image analysis algorithms. An important step towards this goal is to make program code publicly available. The Python code underlying our current manuscript can be found at https://github.com/MedVisBonn/DrusenSegmentation-ModifiedChen.

## Supplementary information

Supplementary information.
