# Author Correction: Replication and Refinement of an Algorithm for Automated Drusen Segmentation on Optical Coherence Tomography

**DOI:** 10.1038/s41598-021-94752-x

**Published:** 2021-07-19

**Authors:** Maximilian W. M. Wintergerst, Shekoufeh Gorgi Zadeh, Vitalis Wiens, Sarah Thiele, Steffen Schmitz-Valckenberg, Frank G. Holz, Robert P. Finger, Thomas Schultz

**Affiliations:** 1 Department of Ophthalmology, University Hospital Bonn, Ernst-Abbe-Str. 2, 53127 Bonn, Germany; 2 Department of Computer Science, University of Bonn, Endenicher Allee 19a, 53115 Bonn, Germany; 3 Department of Medical Biometry, Informatics and Epidemiology, University of Bonn, Bonn, Germany; 4 TIB Leibniz Information Centre for Science and Technology, Hannover and Fraunhofer IAIS, St. Augustin, Germany; 5 Bonn-Aachen International Center for Information Technology, University of Bonn, Endenicher Allee 19a, 53115 Bonn, Germany

Correction to: *Scientific Reports* 10.1038/s41598-020-63924-6, published online 30 April 2020

The Acknowledgements section in the original version of this Article was incomplete.

“This research was supported by the Else Kröner-Fresenius Foundation/German Scholars Organization (EKFS/GSO 16) to RF, the BONFOR GEROK Program, Faculty of Medicine, University of Bonn, (Grant No. O-137.0028) to MW, the GEROK Program, Faculty of Medicine, University of Bonn, (Grant No. O-137.0026) to ST and the German Ministry of Education and Research (BMBF), FKZ 13N10349. The funders had no role in study design, data collection, data analysis, data interpretation, or writing of the report.”

now reads:

“This research was supported by the Else Kröner-Fresenius Foundation/German Scholars Organization (EKFS/GSO 16) to RF, the BONFOR GEROK Program, Faculty of Medicine, University of Bonn, (Grant No. O-137.0028) to MW, the GEROK Program, Faculty of Medicine, University of Bonn, (Grant No. O-137.0026) to ST and the German Ministry of Education and Research (BMBF), FKZ 13N10349. The work of Shekoufeh Gorgi Zadeh was supported by a grant from Deutsche Forschungsgemeinschaft (DFG), grant number SCHM 2966/2-1. The funders had no role in study design, data collection, data analysis, data interpretation, or writing of the report.”

The original Article has been corrected.

