# Supplementary material for: Replication and Refinement of an Algorithm for Automated Drusen Segmentation on Optical Coherence Tomography
Source: Sci Rep. 2020 Apr 30;10:7395. doi: 10.1038/s41598-020-63924-6 (PMC7192932; doi:10.1038/s41598-020-63924-6)
Supplement: Supplementary file 1 — Supplementary information. [file 41598_2020_63924_MOESM1_ESM.pdf]

Replication and Refinement of an Algorithm for Automated Drusen Segmentation on Optical Coherence Tomography

Maximilian W.M. Wintergerst*[1], Shekoufeh Gorgi Zadeh*[2,3], Vitalis Wiens[2,4], Sarah Thiele[1], Steffen Schmitz-Valckenberg[1], Frank G. Holz[1], Robert P. Finger[#1], Thomas Schultz[#2,5]

[1] Department of Ophthalmology, University of Bonn, Ernst-Abbe-Str. 2, 53127 Bonn, Germany

[2] Department of Computer Science, University of Bonn, Endenicher Allee 19a, 53115 Bonn, Germany

[3] Department of Medical Biometry, Informatics and Epidemiology, University of Bonn

[4] TIB Leibniz Information Centre for Science and Technology, Hannover and Fraunhofer IAIS, St. Augustin, Germany

[5] Bonn-Aachen International Center for Information Technology, University of Bonn, Endenicher Allee 19a, 53115 Bonn

*contributed equally

[#]these authors jointly supervised this work

Short title: Algorithm Replication and Refinement on OCT

Supplementary materials and methods

Replication of Chen et al. algorithm

In the algorithm proposed by Chen et al.[1], an anisotropic bilateral filter is applied on the input B-scan, with a window size of 7x19 pixels to account for the stretch in the horizontal direction of B-scans. In our work, we adapted the window size to 5x15 pixels to match the horizontal stretch in our dataset. In order to detect the retinal pigment epithelium (RPE) and ellipsoid zone, a threshold *t*, which is computed using the cumulative histogram, is applied on the denoised B-scan. The left and middle section of **Supplemental Figure 1** show the B-scan before and after thresholding at *t*. Since the RNFL layer is bright as well, it will also be detected by threshold *t*. In order to remove it, the denoised image is thresholded at 0.3**t*. After this, regions of size less than 50 pixels are removed from the thresholded image. The RNFL is then estimated using the first pixel that belongs to the foreground pixels in each column.

For estimating the RPE layer, after thresholding the denoised image at *t*, regions smaller than 50 pixels and all foreground pixels in a neighborhood of 20 pixels within the RNFL layer are removed from the mask. For further clean up, the lower boundary of the RPE region is estimated using the last foreground pixel in each column. Any foreground pixel that falls outside of a 20-pixel neighborhood of the lower boundary is removed. Finally, the RPE layer is estimated by fitting a 3rd degree polynomial to the points at the center of the RPE region (right section **Supplemental Figure 1**). Drusen were defined as the area between the RPE fitted curve as the upper and the ideal RPE estimation as the lower boundary with a minimum drusen height of 2 pixels (**Supplemental Figure 2**). After detecting drusen per B-scan, the en face OCT image was used for a false-positive- elimination-step as proposed by Chen et al[1] (**Supplemental Figure 3**).

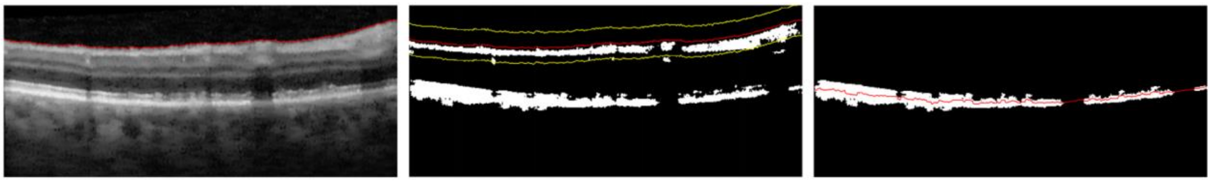

Supplemental Figure 1. Retinal nerve fiber layer removal and retinal pigment epithelium estimation

Estimation of the retinal nerve fiber layer (RNFL) (left), thresholding and narrow band around the estimated RNFL (middle) and estimation of the retinal pigment epithelium centerline (right).

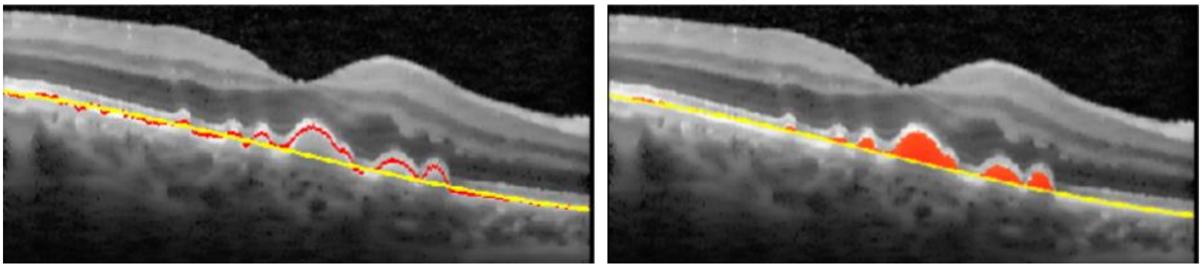

Supplemental Figure 2. Drusen detection

Cropped image of an exemplary B-scan with the retinal pigment epithelium centerline in red and fitted curve in yellow (left) and the resulting drusen segmentation with red domes indicating the detected drusen (right).

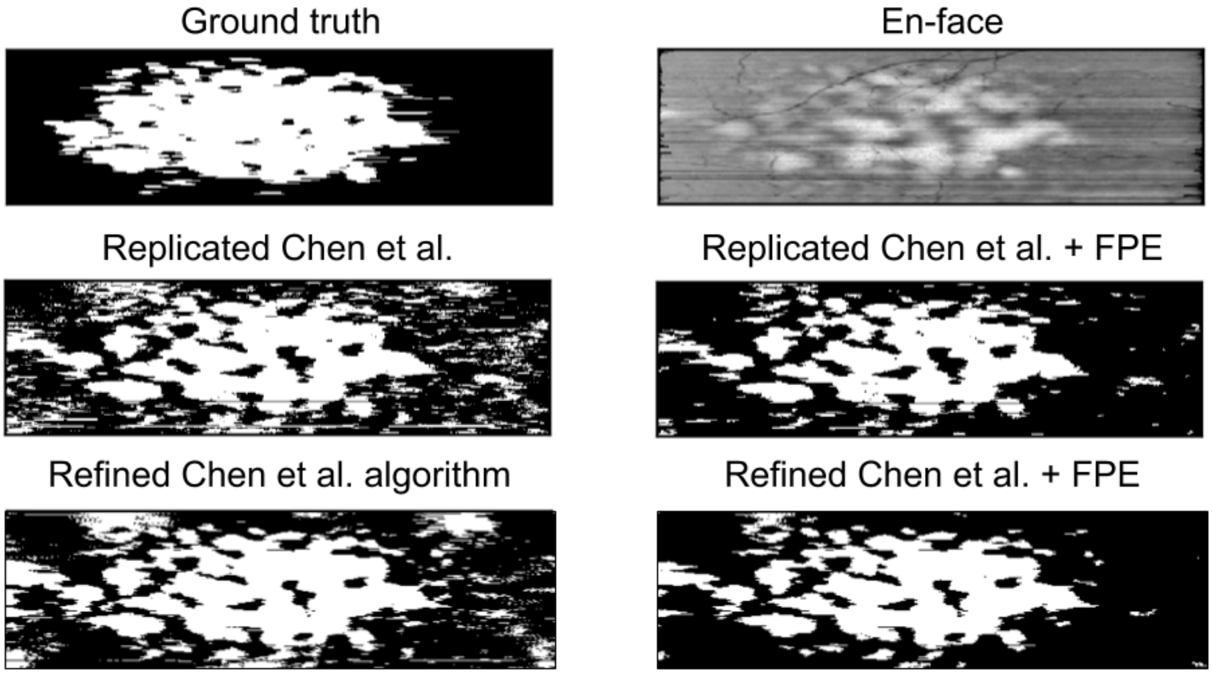

Supplemental Figure 3. False Positive Elimination (FPE)

Segmented drusen are displayed as white against black background with and without FPE. The en-face image is based on the OCT volume scan.

However, **Supplemental Figure 4** (false positives) shows how inclusion of parts of the ellipsoid zone along the RPE can lead to jumps in the estimated RPE layer.

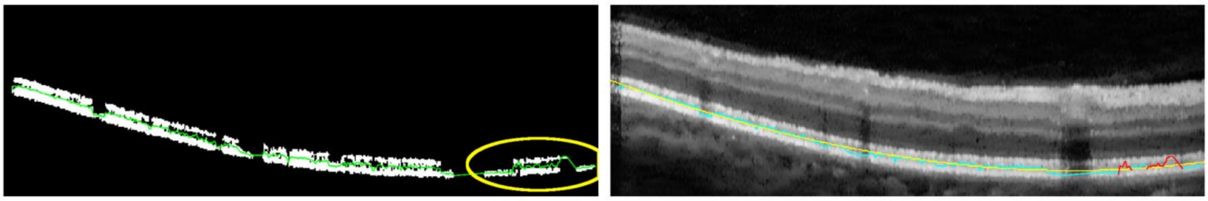

Supplemental Figure 4. Limitation of the replicated algorithm: false positive drusen

Threshold image and centerline (left) and resulting false positive drusen detection (right).

Refinement of Chen et al. Algorithm

Our proposed refinement of Chen et al. algorithm consists of four refinement steps and replacement of the bilateral filter with the multi-scale anisotropic fourth-order diffusion (MAFOD) filter [2].

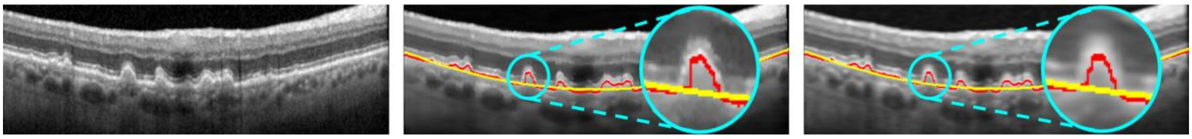

Supplemental Figure 5. Replacing bilateral filter with MAFOD filter

Input B-scan (left), filtered with bilateral filter (middle), and filtered with MAFOD filter (right). MAFOD filter preserves or enhances center-line's intensity value with respect to its neighboring pixels, which results in a more accurate binary estimation of the RPE layer, and consequently a more natural shape of the final layer segmentation.

The MAFOD filter was developed specifically to enhance ridge structures, which appear as bright curves on darker background, e.g., the RPE layer in OCT images. The MAFOD filter automatically detects the scale of the underlying ridge. It then uses the eigenvalues and eigenvectors of the local Hessian matrix, computed at that scale to create a fourth order diffusion tensor, which is utilized to create an anisotropic diffusion effect. The enhancement results in a more accurate estimation of RPE (see **Supplemental Figure 5**). Since within each A scan, shadows similarly affect the brightness of RPE and ellipsoid zone, we address this issue by performing a local histogram equalization within A scans before applying the thresholding. Moreover, in order to reduce speckle noise, Chen et al. work with a filtered version of the OCT scan. Even though we found this noise reduction to be desirable, it also involves a certain amount of blurring that makes it more difficult to separate RPE from ellipsoid zone. We balance the advantages and drawbacks of filtering by incrementally refining the segmentation by Chen et al. in three steps, which are described below, and are based on the original B-scan, as well as two differently filtered versions. As a fourth

refinement, we propose an improved method for extracting the RPE middle axis from the segmentation, in a way that filters out some of the remaining false positives.

In the first refinement step, after initial estimation of RPE using Chen’s method, we use a local histogram equalization with a window of height 80 pixels and width of 1 pixel. Since the goal is to find the RPE in this equalized image, a thresholding with respect to both denoised and locally equalized image is used to detect the brighter regions that are more probable to be part of the RPE. The pixels with values less than 20% of the intensity range in the denoised image, and pixels with values less than 90% of the intensity range in the locally equalized image are set to be background (not part of the RPE). The rest are set as foreground pixels. Using the knowledge that the RPE is thin, far away binary components are set to background. Thus the initial RPE layer estimation that was computed using Chen's method is used to set any pixel outside a 20-pixel neighborhood of the estimated RPE as background. After this step, components smaller than 50 pixels are removed. Then, in order to fill gaps and holes in the estimated area, a dilation of size 2 is performed (see **Supplemental Figure 6**). The newly estimated RPE gives us a more accurate boundary of the RPE area that we will deploy in the further refinement steps.

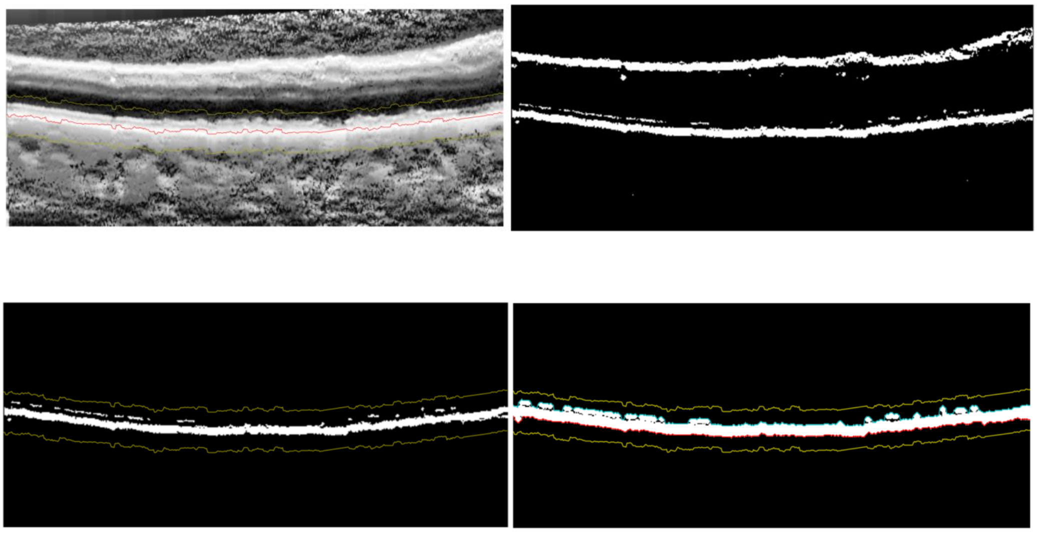

Supplemental Figure 6. First refinement of the retinal pigment epithelium determination

Estimated centerline and local histogram equalization with window size: 80 × 1. (upper left), thresholding with threshold t = 0.9 (upper right), removed RNFL and other bright pixels outside the centerline band (lower left) and double dilation and computation of upper and lower lines (lower right).

In the second refinement step as shown in **Supplemental Figure 7**, a window of size 10 x 1 pixels is used for histogram equalization on the denoised image. Again, a threshold with respect to the denoised image and the locally equalized image is used to binarize the B-scan, i.e., all pixels that have an intensity less than 20% of the intensity range in the denoised image, and pixels with value less than 80% of the intensity range in the equalized image itself, are set to background. In addition, pixels that are outside the estimated RPE area from the first refinement step are removed. Finally, a dilation followed by erosion filter is used to fill in the small holes on the estimated RPE.

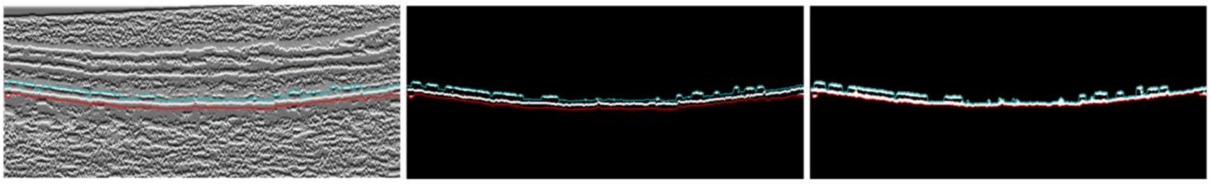

Supplemental Figure 7. Second refinement of the retinal pigment epithelium determination

Local histogram equalization with a window size of 10 × 1 (left), thresholding with t = 0.8 (middle) and an update of the upper and lower lines (right) is shown.

In the third refinement step, the same procedure is repeated on a Gaussian blurred version of the denoised image (see **Supplemental Figure 8)**. We empirically found σ=2 works well on our data-set. The remaining steps are similar to the second refinement step, except that the boundary of the mask computed from the second refinement step are used for removing the non RPE pixels, and only one dilation is performed for filling the gaps.

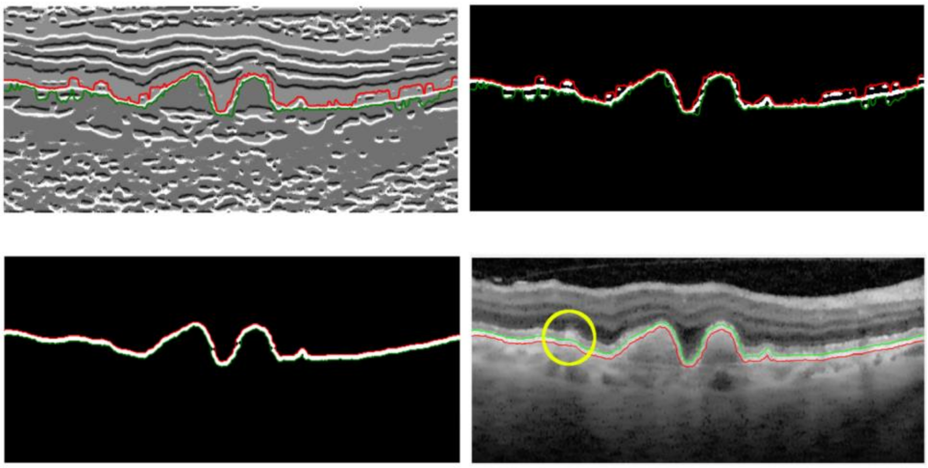

Supplemental Figure 8. Third refinement of the retinal pigment epithelium determination

The resulting image after Gaussian blur and local histogram equalization with a window size of 10 ×1 and red and green lines indicating the upper and lower boundaries estimated from the previous refinement (upper left), thresholding (upper right), the resulting regions produced by shortest path computation (lower left) and an under estimated druse indicated by a yellow circle (lower right).

Drusen detection is based on the middle axis of the RPE. To extract it, Chen et al. simply took the center of the highest and lowest pixels from the RPE segmentation mask in each column of the image, and linearly interpolate in case of gaps. To reduce the impact of small erroneous components that might remain in the final segmentation mask, in the fourth refinement step, we implemented an improved method for estimating the lower and upper boundaries of the RPE from the segmentation mask (see **Supplemental Figure 9**).

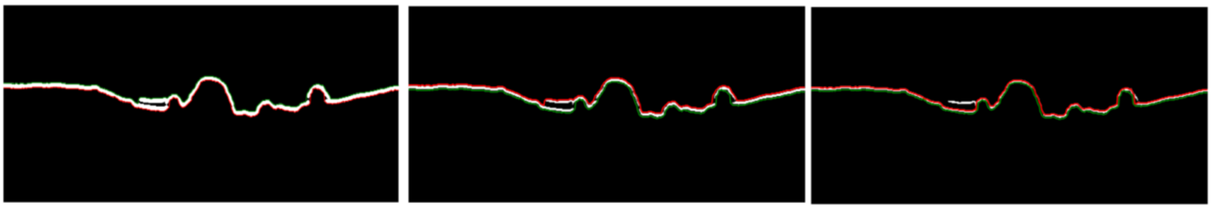

Supplemental Figure 9. Fourth refinement: Improved estimation of the upper retinal pigment epithelial boundary

Result from previous refinement (left), Gaussian, local histogram equalization and thresholding without dilation as in the left image (middle) and the improved upper line estimates (right).

We will explain the procedure in detail for the upper boundary. The lower boundary is found in complete analogy. For finding the upper boundary, our procedure is based on considering the connected components of the segmentation mask, and finding two paths through them, from left to right and from right to left. We only preserve components that are either larger than 200 pixels, or have been used in the paths in both directions. Other components are considered as outliers, and are discarded. From left to right path, we start with the topmost pixel of the leftmost component. From there, always the highest pixel from the current component is picked and is heuristically followed over the upper boundary of the same component until the end is reached. If we reach a column that is not covered by the current component, we move to the topmost component in that column and skip empty columns. After the left-to-right path reaches the image boundary, the last position is used as an initialization point for the search in the inverse direction using the same technique. In the end, we only preserve components that are either very large, or have been used in the paths in both directions. The others are discarded as outliers. As the final step, a third degree polynomial is fit to the upper and the lower boundary, separately. The center-line between the fitted curves is considered as the RPE layer. The python code for both replicated and refinement of Chen et al.'s algorithm can be found at https://github.com/MedVisBonn/DrusenSegmentation-ModifiedChen.

Supplemental Table. Multiple regression analysis for drusen load and presence of geographic atrophy as independent variables and overlap ratio from volumetric computation as dependent variable

	Replicated Chen et al.[1]			Refined algorithm		
	**coefficient**	**p-value**	**95% CI**	**coefficient**	**p-value**	**95% CI**
**Intercept**	9.23	.025	1.19 till 17.28	20.00	< .0001	10.07 till 29.96
**Binned 'medium' relative to 'small' drusen load**	11.18	.019	1.90 till 20.46	13.04	.026	1.57 till 24.51
**Binned 'large' relative to 'small' drusen load**	31.57	< .0001	22.58 till 40.57	35.86	< .0001	24.75 till 46.98
**Presence of geographic atrophy**	-0.035	.992	-6.99 till 6.92	-3.80	.381	-12.41 till 4.80

CI = confidence interval

References

1 Chen, Q. *et al.* Automated drusen segmentation and quantification in SD-OCT images. *Medical Image Analysis* **17**, 1058-1072, doi:10.1016/j.media.2013.06.003 (2013).

2 Gorgi Zadeh, S., Didas, S., Wintergerst, M. W. & Schultz, T. Multi-scale Anisotropic Fourth-Order Diffusion Improves Ridge and Valley Localization. *Journal of Mathematical Imaging and Vision*, 1-13, doi:10.1007/s10851-017-0729-1 (2017).
